# Midlife cardiovascular fitness and dementia: A 44-year longitudinal population study in women

**DOI:** 10.1212/WNL.0000000000005290

**Published:** 2018-04-10

**Authors:** Helena Hörder, Lena Johansson, XinXin Guo, Gunnar Grimby, Silke Kern, Svante Östling, Ingmar Skoog

**Affiliations:** From the Neuropsychiatric Epidemiology Unit (H.H., L.J., X.G., S.K., S.Ö., I.S.), Institute of Neuroscience and Physiology, Sahlgrenska Academy, Centre for Ageing and Health–AGECAP, and Department of Clinical Neuroscience (G.G.), Institute of Neuroscience and Physiology, Sahlgrenska Academy, University of Gothenburg, Sweden.

## Abstract

**Objective:**

To investigate whether greater cardiovascular fitness in midlife is associated with decreased dementia risk in women followed up for 44 years.

**Methods:**

A population-based sample of 1,462 women 38 to 60 years of age was examined in 1968. Of these, a systematic subsample comprising 191 women completed a stepwise-increased maximal ergometer cycling test to evaluate cardiovascular fitness. Subsequent examinations of dementia incidence were done in 1974, 1980, 1992, 2000, 2005, and 2009. Dementia was diagnosed according to DSM-III-R criteria on the basis of information from neuropsychiatric examinations, informant interviews, hospital records, and registry data up to 2012. Cox regressions were performed with adjustment for socioeconomic, lifestyle, and medical confounders.

**Results:**

Compared with medium fitness, the adjusted hazard ratio for all-cause dementia during the 44-year follow-up was 0.12 (95% confidence interval [CI] 0.03–0.54) among those with high fitness and 1.41 (95% CI 0.72–2.79) among those with low fitness. High fitness delayed age at dementia onset by 9.5 years and time to dementia onset by 5 years compared to medium fitness.

**Conclusions:**

Among Swedish women, a high cardiovascular fitness in midlife was associated with a decreased risk of subsequent dementia. Promotion of a high cardiovascular fitness may be included in strategies to mitigate or prevent dementia. Findings are not causal, and future research needs to focus on whether improved fitness could have positive effects on dementia risk and when during the life course a high cardiovascular fitness is most important.

Systematic reviews and meta-analyses of observational studies constantly link physical activity to preserved cognitive functioning and decreased risk for dementia.^1–3^ These studies are limited by reliance on self-reported physical activity and not objectively assessed fitness. Thus, it remains unclear whether the association between physical activity and dementia is mediated by social and cognitive stimulation rather than by level of physical fitness. Furthermore, most studies are conducted in people >60 years of age at baseline, and few have a follow-up of >20 years (mean follow-up 3–7 years), making causal inferences difficult.^4–6^

Aerobic exercise programs aiming at improving cardiovascular fitness seem to have moderate effects on cognitive function among healthy older person.^5,7^ However, current data from randomized controlled trials (RCTs) are insufficient to show that these improvements are due to improved cardiovascular fitness.^5^

Presently, no RCTs and very few long-term prospective studies have been able to relate fitness to dementia incidence. The US Cooper Center Longitudinal Study recently reported that a high midlife fitness, assessed by a maximal treadmill test, was associated with lower risk of developing dementia over a mean follow-up period of 24 years.^8^ Furthermore, 1 large register study among men in Sweden reported that low cardiovascular fitness, assessed with a bicycle ergometer test at 18 years of age, was associated with an increased risk of early-onset (<60 years) dementia.^9^ This is interesting because the etiology of early-onset dementia is supposed to have strong genetic components. Finally, 1 population study from Finland found that poor self-rated fitness in mid to late life was associated with increased dementia risk over 25 years of follow-up.^10^ Thus, there is a need for studies that examine objective fitness before old age with follow-up of dementia until very old age.

Midlife has been suggested as a “sensitive period” for the effect of cardiovascular risk factors on dementia.^11,12^ We therefore tracked dementia incidence for a period of 44 years among women enrolled in the Prospective Population Study of Women (PPSW) who performed a test of maximal cardiovascular fitness in midlife.

## Methods

The study is part of the PPSW, which was initiated in 1968.^13^ Women born in 1908, 1914, 1918, 1922, and 1930 were systematically sampled from the Swedish Population Register on the basis of specific birth dates. Among those sampled, 1,462 women were examined (participation rate 90%). The details and procedures for the examination of the original sample have been described elsewhere.^13^ A systematic subsample (born on the sixth day of uneven months, e.g., January, March, etc) were admitted to an exercise test, and 191 took part (response rate 81%): 29 who were 38 years, 41 who were 46 years, 37 who were 50 years, 47 who were 54 years, and 37 who were 60 years of age.^14^ Follow-ups for dementia diagnoses were performed in 1974 to 1975 (n = 174, 8 had died, 9 refused), 1980 to 1981 (n = 147, 20 had died, 24 refused), 1992 to 1993 (n = 99, 57 had died, 35 refused), 2000 to 2001 (n = 68, 92 had died, 31 refused), 2005 to 2006 (n = 46, 118 had died, 27 refused), and 2009 to 2010 (n = 13, 151 had died, 27 refused). Cases of dementia were also identified via the Swedish Hospital Discharge Register until December 2012.

Participants in the exercise test did not differ from the total sample in age or in cumulative dementia incidence (23.0% vs 22.1%, *p* = 0.780).

### Standard protocol approvals, registrations, and patient consents

The Ethics Committee of the University of Gothenburg approved the study. All women gave informed consent to participate in accordance with the provisions of the Declaration of Helsinki.

### Work capacity

Cardiovascular fitness was tested at baseline in 1968 by a stepwise-increased ergometer cycling test until exhaustion that was supervised by a physician. Details on the full procedure and exclusion criteria have been described previously.^14^ Briefly, after initial submaximal tests of 6 minutes on 200 kilopond m/min (32 W) and 400 kilopond m/min (64 W), the test was interrupted for 5 minutes before the women were brought to maximal workload. The level of maximal workload was chosen on basis of the results from the preceding submaximal test with the aim of achieving an approximate working time of 6 minutes before voluntary fatigue. If the person had not reached her limit of exhaustion, the workload was increased by an additional 50 to 100 kilopond m/min toward the end of the test. During the period of maximal work, heart rate and ECG were registered every minute, blood pressure was registered after 1 and 2 minutes, and respiratory frequency and perceived exertion according to the Borg-scale^15^ were noted after 3 minutes and then every minute. The maximal exercise test aimed at arriving at maximal subjective exhaustion as indicated by the Borg scale^15^; altogether, 93% perceived their maximal load as strenuous (scale point ≥15) and half of the participants as very, very strenuous (scale point 19–20).^14^ The term peak workload is used here because no objective criteria were used for reaching the maximal workload, corresponding to maximal oxygen uptake.

Among 20 women, the test was interrupted during the submaximal test because of changes in ECG (n = 6), too high blood pressure (n = 3), claudication (n = 2), chest pain (n = 1), insufficient cooperation (n = 2), or other reasons (n = 6).^14^ For analytic purposes, these women were categorized as having low fitness. The main results did not change when these persons were excluded.

### Neuropsychiatric examinations and dementia diagnosis

The neuropsychiatric examinations were performed by psychiatrists in 1968 to 1969, 1974 to 1975, 1980 to 1981, and 1992 to 1993 and by experienced psychiatric research nurses in 2000 to 2003, 2005 to 2006, and 2009 to 2010. The examinations were semistructured and included psychiatric interviews, observations of mental symptoms, neuropsychiatric tests, and close informant interviews.^16^

The diagnosis of dementia was based on information from psychiatric examinations, close informant interviews, medical records, and the Swedish Hospital Discharge Registry, as described in detail previously.^16^ For participants in the neuropsychiatric examinations, dementia diagnoses were made by geriatric psychiatrists after reviewing information from both neuropsychiatric examinations and the close informant interview. The diagnosis was made if the participant had dementia according to both sources of information or if there was clear evidence of dementia from 1 source and subthreshold symptoms in the other.

For individuals lost to follow-up, dementia diagnoses were based on information from medical records evaluated by geriatric psychiatrists in consensus conferences and from the Swedish Hospital Discharge Register. The latter provided diagnostic information until December 2012 for all individuals discharged from hospitals on a nationwide basis since 1978.^17^ We have previously reported that the Hospital Discharge Register detects 44% of persons diagnosed at the examinations.^18^

See supplemental data on dementia diagnosis in appendix e-1 (links.lww.com/WNL/A330).

### Confounders

Potential covariates were chosen on the basis of previous research^4^ and biologically relevant variables at the baseline examination in 1968. Education was dichotomized as compulsory (6 years for those born in 1908–1922, 7 years for those born in 1930) or more than compulsory. Smoking was classified as current/ex-smoker vs never smoker. Physical activity during leisure and occupation was assessed according to a slightly modified version of the 4-level Saltin-Grimby scale.^19,20^ Level 1 (almost completely inactive) was classified as physical inactivity. Wine consumption was dichotomized as never drinker or drinker. Hypertension was defined as systolic blood pressure ≥140 mm Hg, diastolic blood pressure ≥90 mm Hg, and/or taking antihypertensive medication. Body height was measured to the nearest centimeter and weight to the nearest 0.1 kg. Body mass index was calculated as kilograms per meter squared. Serum cholesterol and triglyceride levels were assessed after an overnight fast. Diabetes mellitus was self-reported and defined as diagnosis told by a physician or being on antidiabetic therapy (insulin and/or tablets). History of myocardial infarction and angina pectoris was self-reported and defined as a diagnosis told by a physician. The diagnosis of stroke was based on information from participants and key informants, the Swedish Hospital Discharge Registry, and hospital medical records.

### Statistics

Incidence proportions of dementia are presented as cumulative incidence. Differences between fitness groups were analyzed with the χ^2^ test for dichotomous variables and 1-way analysis of variance for continuous variables. We calculated Cox proportional hazards models with all-cause dementia as the outcome and fitness as the predictor. Person-years were calculated from date of baseline examination to (1) time of dementia onset, (2) date of death according to the Swedish Population Register, or (3) December 31, 2012, for all other participants.

For analytic purposes, fitness was described as follows: the crude peak workload categorized into quintiles, but because the 3 middle groups had very similar incidence of dementia, analyses were performed with peak workload categorized into low (≤80 W or interrupted at submaximal workload), medium (88–112 W), and high (≥120 W) fitness; and peak workload/body weight transformed into stanine scores and categorized as low (stanine score 1–3 or interrupted at submaximal workload), medium (stanine score 4–6), and high (stanine score 7–9) fitness.

In model 1, we included age and body height as confounders. In model 2, further confounders were included if bivariate associations in logistic regressions had values of *p* < 0.20 with all-cause dementia (i.e., serum triglycerides *p* < 0.001, smoker *p* = 0.18) or with fitness (i.e., hypertension *p* < 0.001, wine consumption *p* = 0.008, physical inactivity *p* = 0.161, income *p* = 0.010).

All analyses were done with IBM SPSS Statistics 22 (IBM, Armonk, NY). Tests were 2 sided, and the level of significance was set to *p* < 0.05.

## Results

The mean peak workload at the ergometer cycling test in 1968 was 103 (SD 21) W. The midlife characteristics of the study population are presented in table 1. Women with high fitness more often had their own income and higher wine consumption and less often had hypertension compared to those with medium or low fitness. Mean age at death was 80.4 years, and 15% were still alive at the end of the study. We found no statistical difference between the groups in age at death or survival.

**Table 1:**
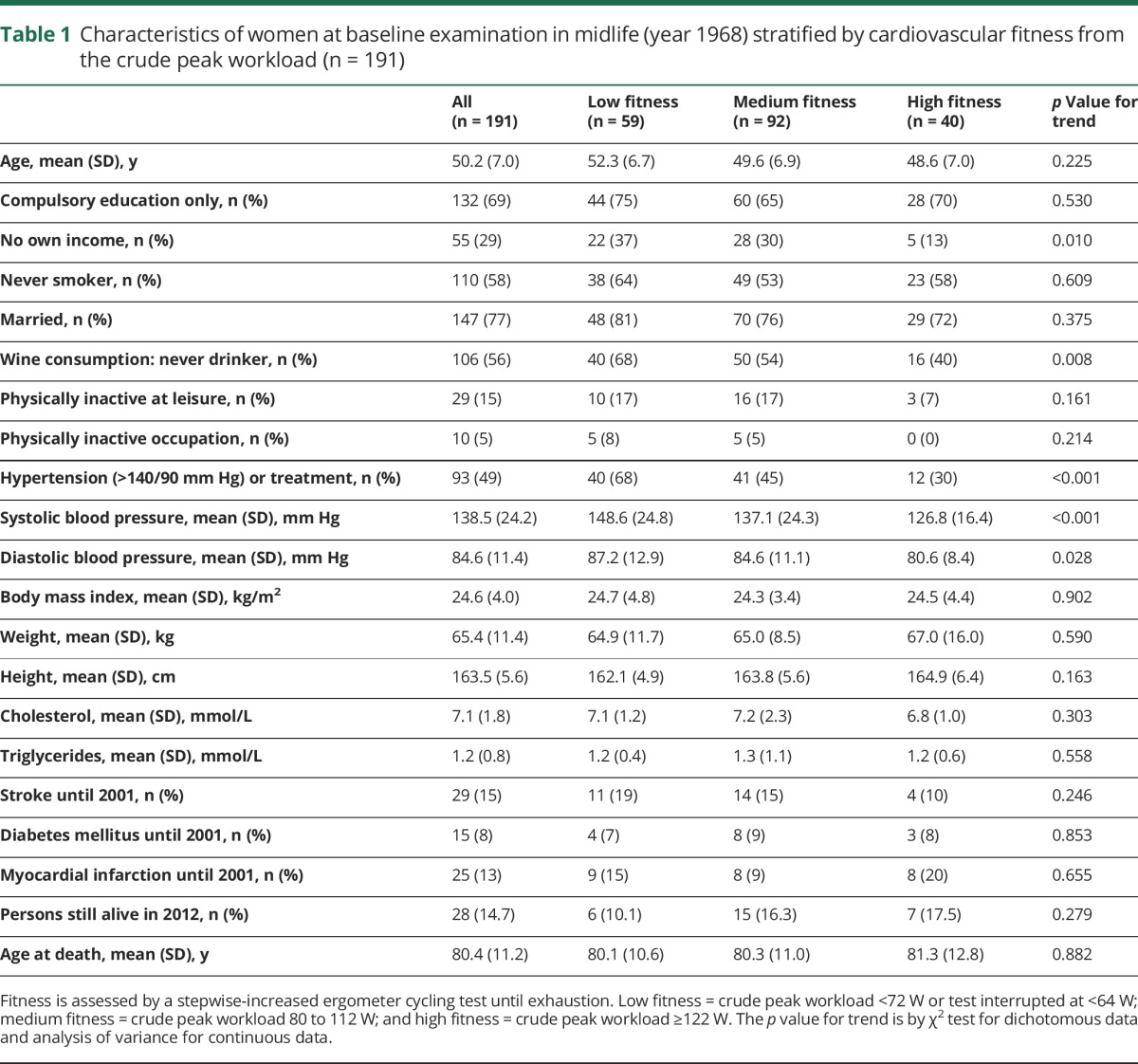
Characteristics of women at baseline examination in midlife (year 1968) stratified by cardiovascular fitness from the crude peak workload (n = 191)

In total, 44 women (23.0%) developed dementia during 5,544 person-years of follow-up from 1968 to 2012. The mean follow-up period was 29 years. Diagnoses included 20 cases of pure Alzheimer dementia, 8 of vascular dementia, 12 of mixed dementia, and 4 of other dementias. Altogether, 28 cases of dementia were diagnosed on the basis of information from the examinations, and another 16 (36%) were diagnosed from registers and case records. The mean time to dementia onset from midlife examination was 29.0 years, and the mean age at dementia onset was 80.5 years.

Table 2 shows the relation between peak workload and cumulative dementia incidence. It is noteworthy that the dementia incidence among those who interrupted the test at submaximal workload was 45%.

**Table 2:**
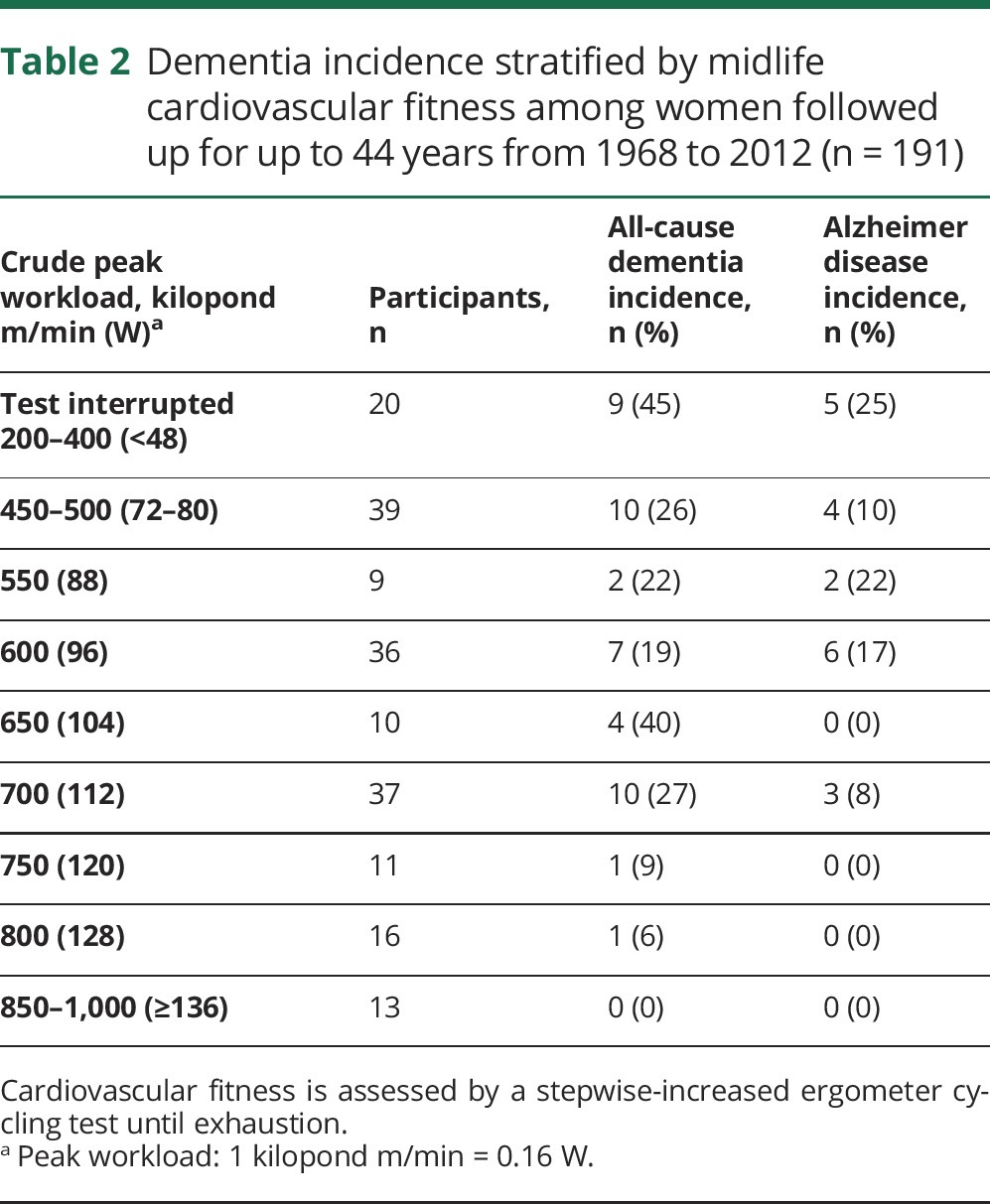
Dementia incidence stratified by midlife cardiovascular fitness among women followed up for up to 44 years from 1968 to 2012 (n = 191)

When categorized into 3 fitness groups based on the peak workload, the cumulative incidence of all-cause dementia was 32% for low, 25% for medium, and 5% for high fitness. Similar results were seen for peak workload/body weight (table 3).

**Table 3:**
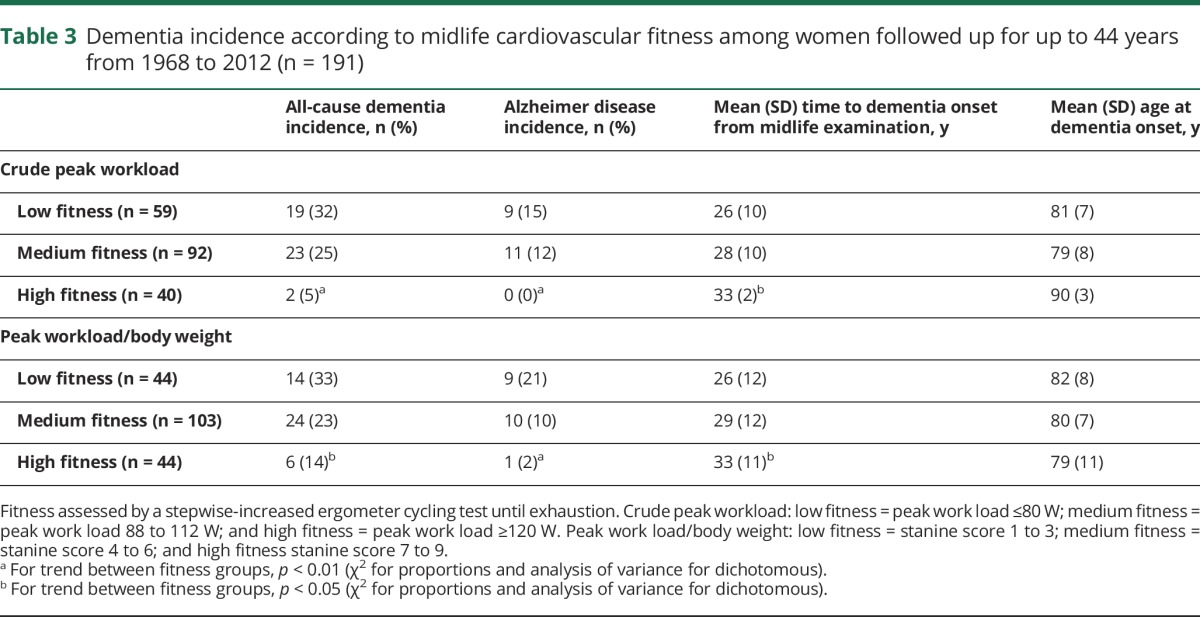
Dementia incidence according to midlife cardiovascular fitness among women followed up for up to 44 years from 1968 to 2012 (n = 191)

The mean time to dementia onset was 5 years longer for those with high compared to those with medium peak workload. The mean age at dementia onset was 11 years higher among those with high peak workload compared to those with medium peak workload (table 3).

Compared to medium peak workload, the adjusted hazard ratio for all-cause dementia was 0.12 (95% confidence interval [CI] 0.03–0.54) among those with high peak workload and 1.41 (0.72–2.79) among those with low workload (table 4).

**Table 4:**
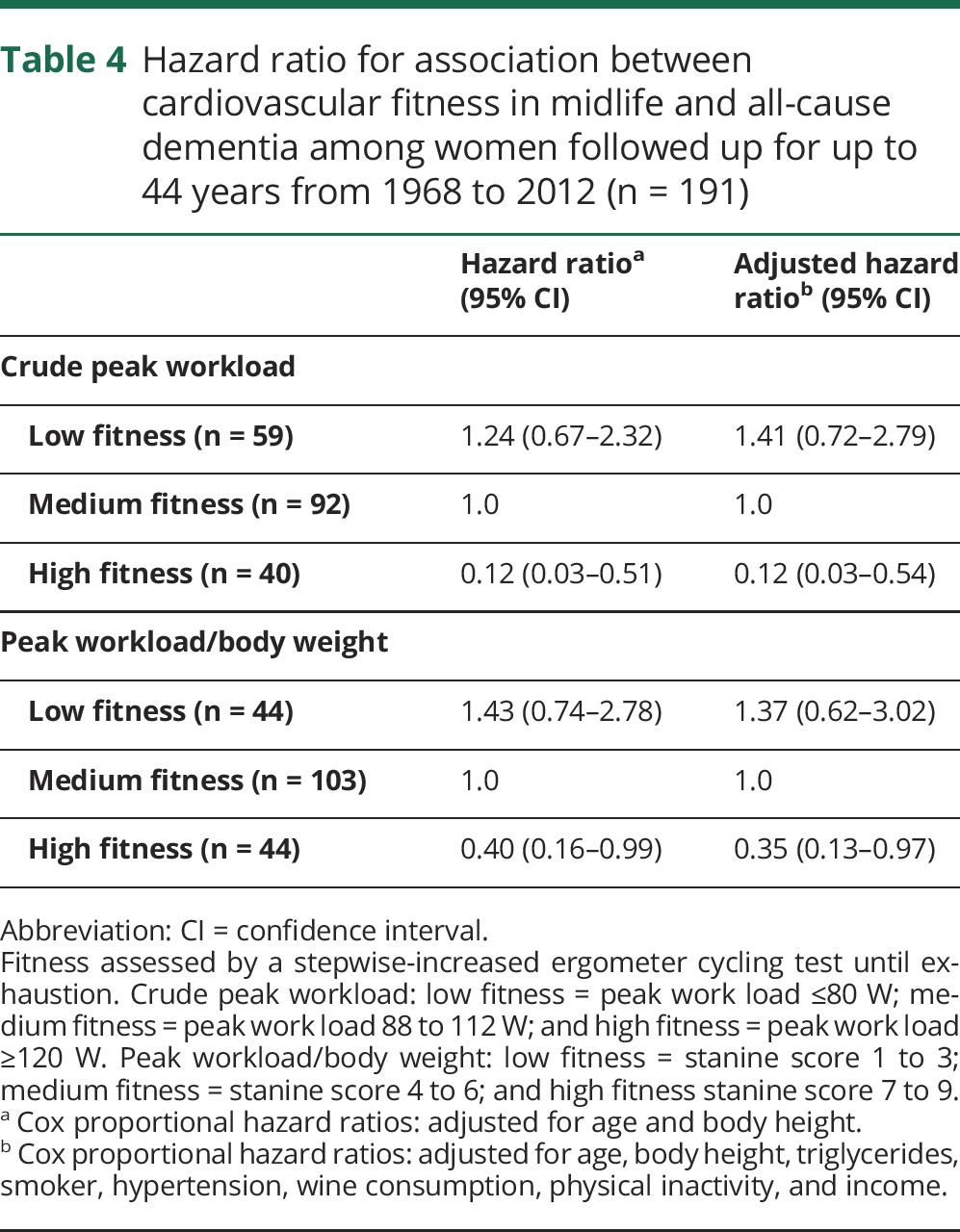
Hazard ratio for association between cardiovascular fitness in midlife and all-cause dementia among women followed up for up to 44 years from 1968 to 2012 (n = 191)

Compared to medium peak workload/body weight, the adjusted hazard ratio for all-cause dementia was 0.35 (95% CI 0.13–0.97) for those with high fitness and 1.37 (95% CI 0.62–3.02) for those with low fitness.

To minimize the influence of incipient dementia on associations between fitness and dementia, we reanalyzed the data excluding those with dementia onset before 70 years of age and dementia onset before the years 1992 and 2000. This did not change the associations (data not shown).

## Discussion

We found that high cardiovascular fitness in midlife was associated with decreased risk of dementia in a population of women followed up for up to 44 years. High compared to medium fitness decreased the risk of dementia by 88%.

The most pronounced risk reduction was seen among participants with the highest fitness. The 3 previous longitudinal studies on fitness and dementia reported a dose-response relation. The US study, which assessed fitness with a maximal treadmill test, found a decreased dementia risk for every fitness quintile. Similar to our study, the lowest risk was seen among those with highest fitness.^8^ On the other hand, the large register study on Swedish men, which assessed fitness according to a bicycle ergometer test at 18 years of age, found an increased risk of early-onset dementia (<60 years) for those with medium fitness compared to those with high fitness and further increased risk for those with low fitness.^9^ The Finnish study, which used a single question of self-rated fitness, found primarily an increased dementia risk among those with poor fitness.^10^ A possible dose-response relation between fitness and dementia risk needs to be further investigated.

We found a very high dementia incidence among those for whom the bicycle test had to be interrupted at submaximal workload. This indicates that adverse cardiovascular processes might be going on in midlife that seem to increase the risk for dementia.

The risk reduction of high fitness on dementia was stronger for the crude peak workload than for peak workload/body weight. This is similar to studies on all-cause mortality in which obese fit individuals have a mortality risk similar to that of normal-weight fit individuals.^21^ This highlights the need for fitness-driven, rather than weight loss–driven, approaches.

Fitness and physical activity are related but not identical.^22^ The hazard ratio in our study was stronger than those reported for physical activity.^2,23^ This is also reported in relation to cardiovascular disease,^22^ indicating that cardiovascular fitness is a more valid measure or that high fitness per se is a stronger protective factor than physical activity. It needs to be emphasized that fitness has a strong genetic component.^24^ Genotype may also modify the association between fitness and dementia. However, evidence is mixed regarding the modifying effect of the *APOE* ε4 allele, the main genetic risk factor for dementia.^1,10^ We had data on genes for only a subsample and cannot draw any conclusions about the impact of *APOE* ε4 on the relation between fitness and dementia. Certain time periods across the life course might be especially important for the effect of cardiovascular fitness. Factors early in life might increase brain reserve, which moderates the expression of brain damage and age-related changes.^25^ Several dementia-prevention RCTs are on the way, all of which target older persons. One is the multidomain Finnish Geriatric Intervention Study to Prevent Cognitive Impairment and Disability (FINGER) study, which targets older persons with cardiovascular risk factors.^26^ This study reported promising results on cognition after 2 years. Another study targeted sedentary older persons and included moderate-intensity aerobic (walking) and strength training,^27^ but it found no effect on cognition after 2 years. Recently, a 6-year multidomain intervention reported no effects on dementia incidence.^28^ Future intervention studies are needed that target whether the actual improvement in cardiovascular fitness (and muscle strength) is the pathway between physical activity and cognitive functioning.^7^ In practice, it will take a very long time to have RCTs that examine the effect of improved midlife (or childhood) fitness on dementia. Meanwhile, longitudinal observational studies such as ours can provide information.

Several mechanisms might be involved in how fitness reduces dementia risk. These include both indirect effects such as influence on hypertension, hypercholesterolemia, obesity, and diabetes mellitus and directs effects on the brain, with, for example, enhancement of neuronal structures, neurotransmitter synthesis, and growth factors.^1,29^ Our study and the 3 other longitudinal studies on fitness and dementia^8–10^ show similar results in unadjusted analyses and analyses adjusted for indirect effects. This indicates that direct effects on the brain need to be further investigated. In line with this, a recent study found that lower cardiovascular fitness was associated with smaller brain volume 2 decades later.^30^ The brain regions that seem most influenced by physical activity are those that are also vulnerable to age-related changes and early pathologic changes in Alzheimer disease such as the hippocampus.^31^ Further research on long-term direct effects of fitness on brain structure is needed to improve strategies for dementia prevention.

Major strengths of our study are the objective assessment of fitness, the fact that baseline examinations were carried out in midlife, the 44 years of follow-up, that the dementia diagnosis was made by neuropsychiatrist according to extensive examinations, the population-based sample, and the extensive collection of potential confounders. However, there are several limitations. First, this study had an observational design; therefore, we cannot draw conclusions on cause and effect. Second, the sample was relatively small, leading to a lack of statistical power and limiting the possibility for subanalyses. Third, the study includes a relatively homogeneous sample of Swedish women. We thus cannot generalize to other populations. In addition, women in the study probably received more medical care than other women because persons in whom we identified pathologic conditions (e.g., hypertension) were referred for medical treatment. Fourth, cumulative dropout is a problem in long-term follow-up studies. While this problem was, to some extent, alleviated by the use of hospital registry data for those lost to follow-up, this probably results in an underestimation of the number of dementia cases. It should be noted that almost all people in Sweden receive hospital treatment within the public health system, and the Swedish Hospital Discharge Register covers the entire country. Fifth, the exercise test in 1968 measured work capacity, not maximal oxygen consumption with expired gas analysis, the gold standard for cardiorespiratory fitness. Sixth, the maximal workload for the women in our study is lower compared to previously reported reference values.^32^ This might be due to different procedures for the exercise test. Seventh, we did not have data on changes in fitness across the life course. Eighth, competing risk may influence the results of a study with long-follow-up because both dementia and low fitness may increase the risk for death. This might result in an underestimation of the association between these conditions. The use of risk-years in the Cox regression analyses partly takes care of competing risk because persons who die earlier will contribute fewer years.

Our findings indicate that high cardiovascular fitness in midlife is associated with decreased risk of dementia. Improved cardiovascular fitness in midlife might be a modifiable factor to delay or prevent dementia. Findings are not causal, and future research needs to focus on whether improved fitness could have positive effects on dementia risk and when during the life course a high cardiovascular fitness is most important.

## Glossary

CI: confidence interval
FINGER: Finnish Geriatric Intervention Study to Prevent Cognitive Impairment and Disability
PPSW: Prospective Population Study of Women
RCT: randomized controlled trial
